# Isotope incorporation in broad-snouted caimans (crocodilians)

**DOI:** 10.1242/bio.20134945

**Published:** 2013-05-20

**Authors:** Stephane Caut

**Affiliations:** Estación Biológica de Doñana, Consejo Superior de Investigaciones Científicas (CSIC), Apartado 1056, E-41080 Sevilla, Spain

**Keywords:** Crocodilian, Diet, Excretion, Discrimination factor, Fractionation, Nitrogen enrichment, Turnover

## Abstract

The trophic ecology and migration of vertebrate species have been increasingly studied using stable isotope analysis. However, this approach requires knowledge on how dietary isotopic values are reflected in consumers' tissues. To date, this information has only been obtained for a handful of ectotherms; in particular, little is known about crocodilians. In this study, diet-tissue discrimination factors (DTDFs) and carbon and nitrogen stable isotope turnover rates were estimated for plasma, red blood cells (RBCs), and muscle obtained from broad-snouted caimans (*Caiman latirostris*). Individuals were fed two different control diets for 189 days. DTDFs for δ^15^N (Δ^15^N) and δ^13^C (Δ^13^C) ranged from −2.24‰ to 0.39‰ and from −0.52‰ to 1.06‰, respectively. Isotope turnover rates in tissues, expressed as half-lives, ranged from 11 to 71 days, with plasma<muscle<RBCs. Δ^15^N was found to be particularly small, even when compared to values found for other ectotherms, a result that may be linked to the unique excretion physiology of crocodilians. These stable isotope incorporation data should help inform future interpretations of isotopic values obtained in the field for this taxon.

## Introduction

Based on the assumption that “*you are what you eat*”, stable isotope analysis is a widespread tool in studies of diet composition, consumer trophic level, and even habitat use and migration (Rubenstein and Hobson, 2004). Since the carbon isotope ratio ^13^C/^12^C changes minimally (∼1‰) as carbon moves through food webs, it is commonly used to evaluate the dietary source of carbon. In contrast, the nitrogen isotope ratio (^15^N/^14^N) in consumers' tissues is typically considered to be enriched by ∼3‰ relative to that in the diet; it is thus commonly used to estimate trophic position. Stable isotope analysis has become an advantageous and complementary tool when characterizing feeding or migration behaviors that are difficult to examine using conventional techniques (e.g. gastric lavage, fecal analysis); it also provides information on the foods that are assimilated, and not just ingested, as well as clarification on how the diet is integrated into tissues over time (Caut et al., 2008).

Over the last 10 years, we have observed an explosion in the number of studies using isotopes to investigate trophic ecology and animal migration in the field. However, we have also become aware of the necessity of conducting laboratory studies that clarify the isotopic incorporation process in different species or under different conditions so as to better interpret these field data. Indeed, two major parameters that are the basis for stable isotope interpretation appear to be highly variable across taxa, tissues, and diets (Caut et al., 2009). These parameters are the turnover rate, i.e. the time it takes for an isotope to be assimilated into consumer tissue, and the discrimination factor, i.e. the difference between the stable isotope composition of a given tissue and that of the diet. Numerous experimental studies have investigated isotope incorporation in mammals, birds, fish, and invertebrates. However, isotopic calibration has only recently been performed for taxa that are difficult to study under controlled conditions, such as cetaceans (Caut et al., 2011) and sharks (Hussey et al., 2012), even though stable isotopes had been previously used to investigate the ecology of these marine groups.

Despite the need for laboratory experiments that evaluate assumptions about stable isotope ecology, few such experiments have been performed on ectothermic terrestrial vertebrates. Indeed, reptilian metabolism and regulatory physiology are distinct, and parameter estimates obtained from validation studies in other taxonomic groups may lead to inappropriate conclusions about these ectotherms. Only eight studies on isotope incorporation in reptiles have been published [tortoise (Murray and Wolf, 2012), freshwater turtles (Seminoff et al., 2007), sea turtles (Seminoff et al., 2006; Reich et al., 2008; Seminoff et al., 2009), snakes (Fisk et al., 2009), lizards (Warne et al., 2010), and alligators (Rosenblatt and Heithaus, 2013)]. Nonetheless, the results of these few studies have been used to interpret field data from these species as well as guide future research and new study questions [e.g. sea turtles (Ceriani et al., 2012)]. In this context, crocodilian taxa are surprisingly understudied; only three recent studies of crocodilian trophic ecology employed stable isotopes (Rosenblatt and Heithaus, 2011; Radloff et al., 2012; Wheatley et al., 2012). Given these species' complex habitats and nocturnal foraging behavior, stable isotopes may present a considerable advantage over traditional methods, such as stomach content analysis and feeding observations, when analyzing crocodilian diets. Isotopic analysis is routinely used to study the diet and habitat use of other difficult-to-study aquatic taxa [e.g. sea turtles (Ceriani et al., 2012), marine mammals (Newsome et al., 2010), and sharks (Hussey et al., 2012)].

Because the use of stable isotope analysis to address crocodilian ecology is likely to expand, there is a need to better estimate isotope incorporation in these taxa to help and encourage future crocodilian research. In this study, I experimentally quantified diet-tissue discrimination factors (DTDFs) and carbon and nitrogen stable isotope turnover rates in plasma, red blood cells (RBCs), and muscle obtained from broad-snouted caimans (*Caiman latirostris*).

## Materials and Methods

### Experimental design

**Fig. 1.**
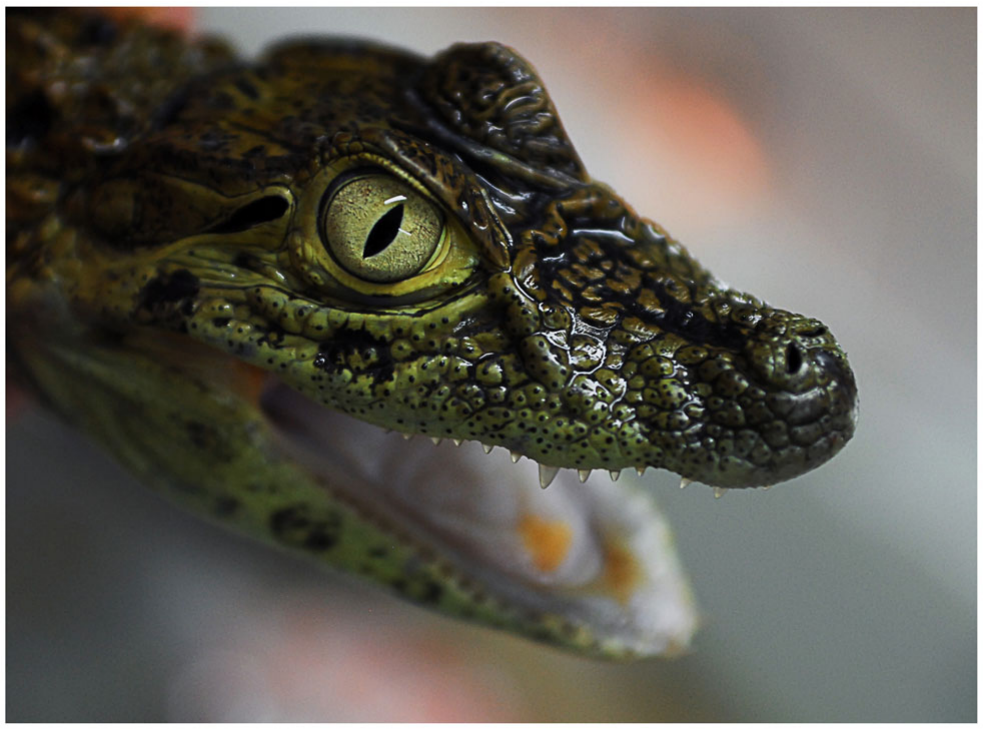
Juvenile broad-snouted caiman (*Caiman latirostris*) studied in captivity at the Alligator Bay Zoological Park (Mont-Saint-Michel, France). The most notable physical characteristic is its broad snout adapted to rip through the dense vegetation while foraging for food. Photograph reproduced with the kind permission of the copyright holder, Samuel Decout.

Twenty-three broad-snouted caimans (*Caiman latirostris*, length 47.5±1.1 cm and mass 339.7±25.4 g, Fig. 1) were studied in captivity at the Alligator Bay Zoological Park (Mont-Saint-Michel, France). All caimans were born in the park and were 10 months old. Prior to the start of the experiment the caimans and their mothers were fed a diet of adult chickens (*Gallus* sp.) that had not started to lay eggs. Three individuals died at nine months, and their muscle tissues were sampled to estimate isotopic ratios at T_0_. To identify individuals during the experiment, I implanted microchips under the caudal skin (*Virbac* microchips, Carros, France). Caimans were randomly assigned to one of two dietary treatments: roach fish diet (R, *Rutilus rutilus n* = 11) or baby chicken diet (C, *Gallus sp. n* = 12). These two diets were from the commercial farm St Laurent (La Chapelle Saint Laurent, France; http://www.st-laurent.fr) and both were from the same frozen stock, bought at the beginning of the experiment. Individuals were fed 50 g three times per week. At 97 days, six caimans were switched from the R diet to C diet (R_97_C_92_) and six were switched from the C diet to R diet (C_97_R_92_); they consumed the new diet for an additional 92 days. Six (C diet; C_189_) and five (R diet; R_189_) caimans in each treatment continued on the same diet for the same amount of time (92 days). To implement this experimental design, caimans were placed in 4 large aquariums (one for each treatment) of the same configuration (100×200×100 cm in size, with UV light, filtered water, and a rock) and with the same physicochemical conditions.

Blood samples were taken and total length and body mass were measured at the start of the experiment (T_0_), at five days (T_5_), and every 15 days thereafter. Blood was obtained from the cranial sinus using blood-collection kits. The blood sample was immediately separated into red blood cells (RBCs) and plasma components by centrifugation. At days 97 and 189 of the experiment, tail muscle samples were collected for all individuals (biopunch, diameter 2 mm followed by a reparative stitch). The isotopic value of each diet was quantified; samples were randomly taken from the stock throughout the experiment. All samples were kept at −20°C until isotopic analysis.

### Isotopic analyses

Caiman tissues (plasma, RBCs, and muscle) and diet samples (roach fish and chick) were freeze-dried and ground to a fine powder. Lipid extraction was performed on diet samples by rinsing them with a 2:1 chloroform:methanol solvent and then drying them at 60°C for 24 h to remove any residual solvent. The extraction of lipids from tissue samples was unnecessary because the lipid component in these tissues is generally minor and less than 3.5 [C/N_PLASMA_ = 1.93±0.03; C/N_RBC_ = 2.26±0.03, *n* = 380; C/N_MUSCLE_ = 3.31±0.02, *n* = 47 (Post et al., 2007)]. Bulk tissue samples (lipids not extracted) and lipid-extracted diet samples were then analyzed for stable isotopes. Isotopic analyses were performed on 1 mg subsamples of the homogenized materials that had been loaded into tin cups.

Stable carbon and nitrogen isotope measurements were carried out using a continuous flow isotope ratio mass spectrometer (Optima, Micromass, UK) coupled to a C-N-S elemental analyser (Carlo Erba, Italy). Stable C and N isotope ratios are expressed as: δ^13^C or δ^15^N =  [(*R*_sample_/*R*_standard_)−1]×1000, where *R* is ^13^C/^12^C or ^15^N/^14^N for δ^13^C or δ^15^N, respectively. *R*_standard_ is the ratio of the international references PDB for carbon and AIR for nitrogen. One hundred replicate assays of internal laboratory standards indicate maximum measurement errors (SD) of ±0.2‰ and ±0.15‰ for δ^13^C or δ^15^N measurements, respectively.

### Isotopic incorporation

The isotope incorporation parameters were calculated for the two constant diet treatments (R_189_ and C_189_). Isotope turnover rates were quantified by fitting the data using a Marquardt non-linear fitting routine (NLIN, SAS) using the following equations:
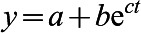
where *y* is δX (^13^C or ^15^N), *a* is the isotopic value approached asymptotically (δX_(∞)_), *b* is the total change in isotopic values after the diets were switched (δX_(∞)_−δX_(t)_), *c* is the turnover rate, and *t* is the time in days since the switch. To find the length of time required for *α* % turnover, the following equation was solved:
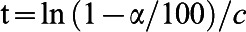
where t is the time in days, *α* is % turnover (e.g. half-lives *α* = 50), and *c* is the turnover rate of the tissue. For muscle, isotope incorporation was quantified using the values at T_0_, T_97_, and T_189_.

Discrimination factors between a consumer (*caiman*) and its food resource (*food*) are described in terms of the difference in delta (δ) values using the Δ notation; DTDF (Δ) = X_(∞)caiman_−X_food_, where X is δ^13^C or δ^15^N. When the model did not fit to convergence for one diet, but the 95% turnover rate for the second diet was shorter than the duration of the experiment (189 days), I calculated the DTDF at T_189_.

### Statistical analyses

I performed factorial ANOVAs to test (*a*) the effect of lipid extraction on the isotopic ratios of the two diet types (roach fish and chick) – values resulting from lipid extraction are noted hereafter as DEL – and (*b*) the effect of body mass on the isotopic values of caiman tissues at T_189_.

Computations were performed using STATISTICA 6.0, and isotope incorporation data were fitted using a Marquardt non-linear fitting routine (NLIN, SAS, Cary, NC, USA). The level of significance for the statistical analyses was set at *P* = 0.05.

## Results and Discussion

**Fig. 2.**
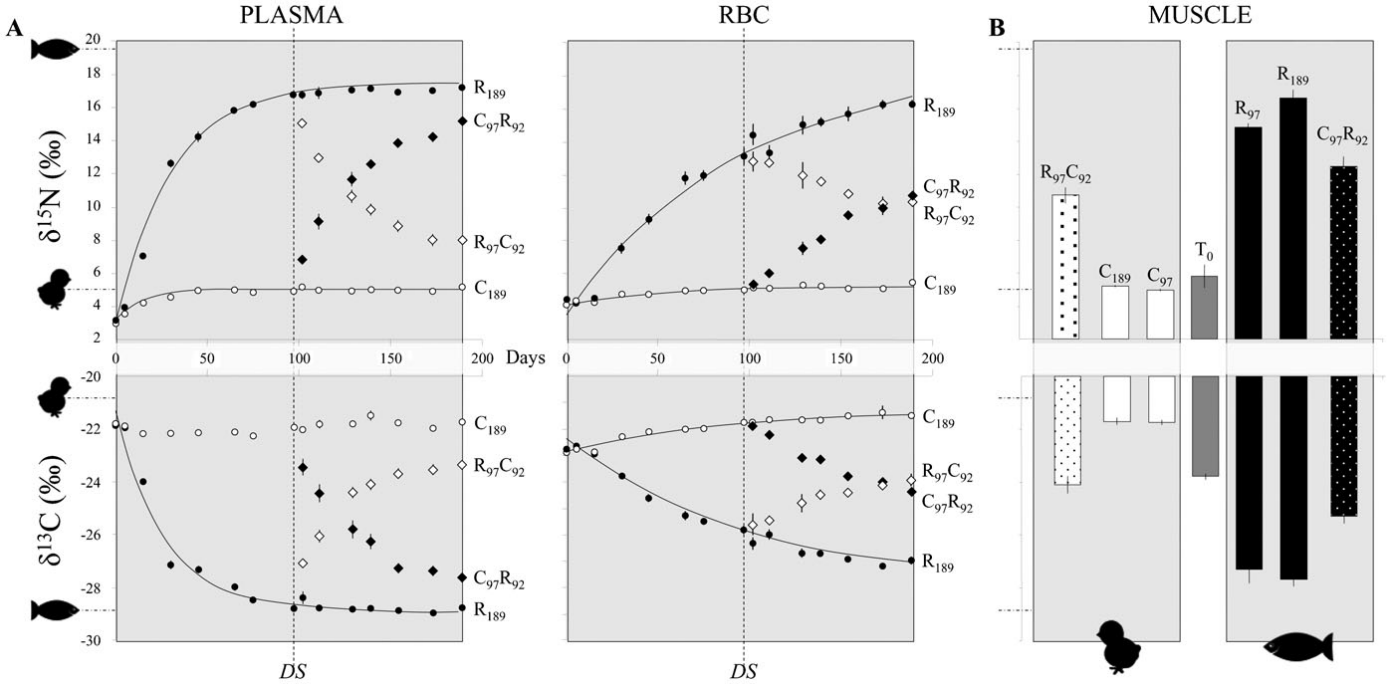
(**A**) Nitrogen and carbon isotopic values (mean±SD) of plasma and red blood cells (RBCs) for the different dietary treatments; (*i*) R_97_C_92_ = switch from roach fish (R) to chick (C) diet at 97 days (Diet Shift, *DS*); (*ii*) C_97_R_92_ = switch from C to R diet at 97 days; (*iii*) R_189_ and C_189_ = constant diet (R or C) for 189 days. Dietary treatments R_97_ and C_97_ represent the first part of the experiment (0–97 days), before the dietary shift occurred (*DS*). Exponential fits are only shown when significant. The mean isotopic values for the two diets are represented by icons (chick and roach fish) on the y-axis. (**B**) Nitrogen and carbon isotopic values (mean±SD) of caiman muscle for the different treatments (chick: C_97_, C_189_, R_97_C_92_; roach fish: R_97_, R_189_, C_97_R_92_) and at the beginning of the experiment (T_0_).

Research has yet to quantify isotope incorporation in crocodilians, which probably explains the almost complete absence of field studies employing isotopic analysis, a technique that is particularly suited to the sampling challenges and ecology of this taxon. In this study, δ^15^N and δ^13^C values of plasma and RBCs in all treatments significantly fit an exponential model; the only exception was the δ^13^C of plasma in the C_189_ group (Table 1A; Fig. 2). Isotope turnover rates (50%) were fastest in plasma [11–21 days], intermediate in muscle [31–43 d], and slowest in RBCs [37–71 d]. These incorporation rates are very similar to those of other reptiles with the same plasma<RBC (Table 2), but greater than those values reported for other similarly sized ectotherms, especially for RBC [e.g. rat t_1/2Blood_ = 25 d (MacAvoy et al., 2006); quail t_1/2Blood_ = 11.4 d and crow t_1/2RBC_ = 30 d (Hobson and Clark, 1992; Hobson and Clark, 1993); or cetaceans t_1/2Blood_∼30 d (Caut et al., 2011)]. As suggested by Murray and Wolf reptile plasma incorporation rates are likely similar to those of other ectotherms (Murray and Wolf, 2012) because plasma proteins are largely synthesized in the liver which have a similar function in most organisms (Tieszen et al., 1983). In contrast, RBCs are in general long-lived in reptiles [e.g. alligator = 1320 d (Cline and Waldmann, 1962); turtle = 300–800 d (Altland and Brace, 1962; Krasil'nikov, 1971)] partially due to their nucleated RBCs (Dessauer, 1970).

**Table 1.**
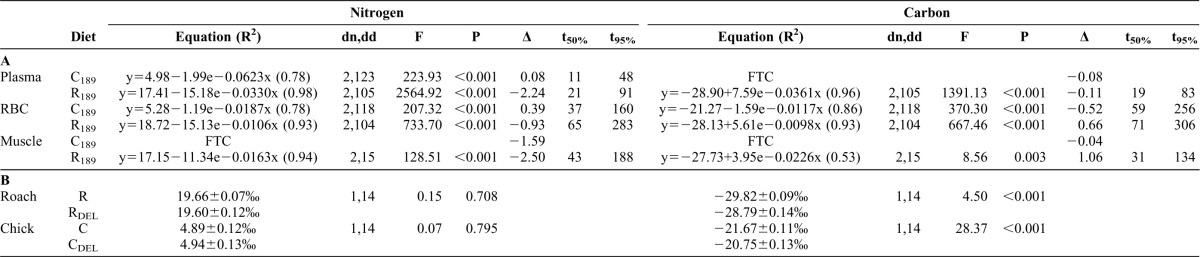
(A) Exponential equations (with R^2^) and statistics of converging models for stable isotope incorporation in the plasma, RBCs, and muscle of caimans kept under controlled conditions. Nitrogen and carbon discrimination factors (Δ in ‰) and turnover rates (t_50%_ and t_95%_) in days in different caiman tissues (δ^13^C calculated from the lipid-extracted diet sample and δ^15^N from the bulk, non-lipid-extracted diet sample). The two constant diet treatments were analyzed (C_189_ and R_189_). When the exponential model failed to significantly converge (FTC) for one diet, but the 95% turnover rate for the second diet was shorter than the duration of the experiment (189 days), I calculated the Δ at T_189_. (B) Effect of lipid extraction on the nitrogen and carbon isotopic values of the two diets (roach fish and chick).

**Table 2.**
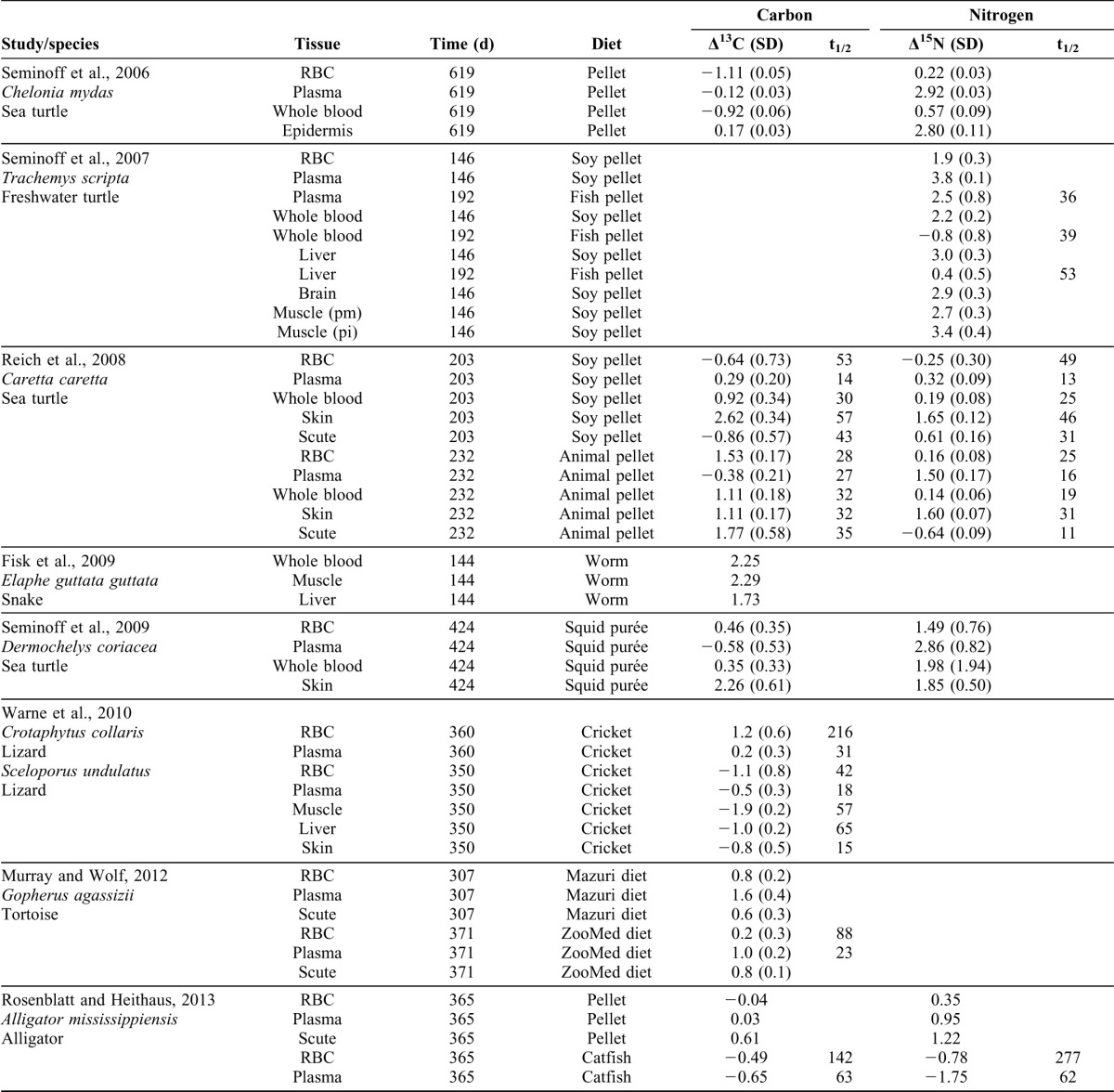
Description of carbon and nitrogen discrimination factors (Δ in ‰, mean±SD) and half-life turnover rates (t_1/2_, in days) for different tissues obtained from a literature search. Time and diet columns list experiment duration (in days) and type of control diet used, respectively.

Our results differ somewhat from those presented in a recent study of alligators (Rosenblatt and Heithaus, 2013). That study reported a higher turnover of carbon and nitrogen (t_1/2_ = 142 d and 277 d, respectively) in RBCs than we found or than is the general trend in reptiles (Table 2). In general, incorporation rates increase when body size decreases (Martínez del Rio et al., 2009; Warne et al., 2010). Rates of incorporation are heavily dependent on tissue-specific protein turnover rates (Carleton and Martínez del Rio, 2005), which tend to be significantly higher as metabolic rate increases (endothermic species have metabolic rates that are seven to ten times those of ectotherms). The juvenile alligators studied by Rosenblatt and Heithaus (Rosenblatt and Heithaus, 2013) were larger than the caimans we studied or the other reptiles whose isotopic turnover rates have been studied (Table 2). Our caimans were younger and they were undergoing more rapid growth than the alligators used in their study and therefore they were incorporating isotopes from the diet into new tissues at a fast rate. Another possible explanation for the difference between our results and those of Rosenblatt and Heithaus (Rosenblatt and Heithaus, 2013) could be the small isotopic amplitude of their diet shift. In our case the isotopic diet shift was very high in ∼15‰ and ∼10‰ for δ^15^N and δ^13^C respectively. Indeed, the exponential model fits the isotopic dynamics better when the isotopic shift is higher and when the frequency of sampling is shorter.

By the end of the experiment, plasma and muscle tissues had achieved equilibrium with the diet, allowing DTDFs to be accurately estimated. Exponential models fitting isotopic incorporation in tissues are extremely sensitive to experiment duration, and it is best to ensure that the length of the experiment is long enough to observe equilibrium. As a result, the constant diet groups (R_189_ and C_189_) were used to estimate the incorporation parameters, a decision whose validity was supported by the dynamics of the treatments in which diets were switched (Fig. 2A). Another important factor is the initial isotopic difference between the study animal and its new diet; the greater the difference, the better the fit of the incorporation model. This fact probably explains the difference in turnover rates between the chick and roach fish diets and the lack of convergence for the exponential model for the chick diet, whose initial isotopic difference was less marked (Table 1A; Fig. 2A).

The use of an accurate DTDF is also highly important, as this parameter has been shown to vary across tissues, species, and dietary isotopic values (Caut et al., 2009). I used lipid-extracted δ^13^C_DEL_ and non-lipid-extracted δ^15^N to calculate isotope incorporation because lipid extraction significantly affected dietary δ^13^C but not δ^15^N (Table 1B). Caiman mass did not significantly affect final δ^13^C and δ^15^N values in any of the tissues (*P*>0.05). Thus, the Δ^13^C observed in caiman tissues, which ranged from −0.52 to 1.06‰ (Table 1A), had the same range as in previous studies of endotherms (for reviews, see Caut et al., 2009; Martínez del Rio et al., 2009) or other ectotherms (Table 2). These similarities could be explained by the fact that discrimination processes are associated with biochemical processes (e.g. Kreb's cycle), which are conserved across taxa and relatively insensitive to fluctuations in biological rates and temperature (Martínez del Rio et al., 2009). In contrast, the Δ^15^N estimates [−2.50 to 0.39‰; Table 1A] do not correspond to those classically predicted by nitrogen trophic enrichment theory (∼3‰). The other study of crocodilians found the same low Δ^15^N estimate in alligators, especially under the catfish diet [−0.65 to 0.28‰ (Rosenblatt and Heithaus, 2013); Table 2]. Although Martínez del Rio et al. predicted a smaller Δ^15^N in growing than non-growing animals (Martínez del Rio et al., 2009), these particularly small estimates are nonetheless surprising. Indeed, previous controlled experiments conducted on juvenile turtles found larger and more positive values in the same tissues [loggerhead turtles, −0.25 to 1.50‰ (Reich et al., 2008); green turtles, 0.22 to 2.92‰ (Seminoff et al., 2006)].

In the literature, the ^15^N enrichment of tissues is often attributed to the preferential excretion of light nitrogen (^14^N). However, Δ^15^N values vary significantly depending on the form in which nitrogenous waste is excreted. The enrichment was least for animals excreting ammonia, intermediate for animals excreting uric acid, and greatest for animals excreting urea (Vanderklift and Ponsard, 2003). The nitrogenous end products excreted by reptiles are more diverse than those of mammals (urea) or birds (uric acid). Indeed, tortoises excrete urea and uric acid, lizards and snakes chiefly excrete uric acid, and crocodilians produce ammonia and uric acid (Singer, 2003). Crocodilian excretion is very species-specific and poorly characterized. Fasting crocodiles and alligators release approximately equal quantities of ammonia and uric acid in their urine. However, when they are fed *ad libitum*, the proportion of ammonia increases, while that of uric acid decreases, and only negligible amounts of urea are produced (Huchzermeyer, 2003). Thus, based on the limited comparison of these crocodilian data with other reptilian data, there may be a potential effect of excretion on DTDFs. This research also underscores the lack of experimental data available on isotope incorporation in reptiles and, more precisely, in crocodilians.

At a finer scale, differentiation in DTDFs was higher between diets (roach fish vs chick) than among tissues: Δ^15^N was more enriched in the chick diet than in the roach fish diet, and the inverse was true for Δ^13^C. Discrimination factors may vary as a result of relationships between DTDFs and dietary isotopic values; Δ^15^N and Δ^13^C decrease as δ^15^N and δ^13^C increase (for a review, see Caut et al., 2009). Indeed, I used two control diets with very different isotopic values to represent the broad range of isotopic values of potential natural prey, because crocodilian species are known to be predators with a wide dietary range (Radloff et al., 2012).

In conclusion, it is difficult to elucidate the trophic ecology of crocodilians because they are aquatic and often nocturnal. Although traditional methods, such as the analysis of stomach contents, provide some sense of the prey taxa being consumed, they can only be used on dead individuals or individuals whose stomachs are invasively lavaged. The results they provide may thus be affected by several sources of bias. More importantly, stomach contents only reveal a snapshot of an animal's total diet. The stable isotope ratios of different tissues can reveal the trophic ecology of a predator, including changes in diet and habitat use, over different time scales (e.g. plasma 3 months, muscle 6 months, or RBCs 1 year). To successfully conserve crocodilians and their aquatic ecosystems, it is important to understand and predict changes in their diet and habitat use, as well as to characterize their capacity to contend with environmental modification. To date, only three studies have used stable isotope analysis to investigate crocodilian trophic ecology (Rosenblatt and Heithaus, 2011; Radloff et al., 2012; Wheatley et al., 2012). We studied rapidly growing caiman juveniles maintained under constant optimal conditions, so our measured rates of incorporation probably represent higher rates for this species than those seen in wild caimans enduring episodic nutritional constraints and growing more slowly (e.g. adults). These estimates of isotope incorporation should encourage future research using this method, which is well suited to the challenges of studying this taxon and which is routinely used in studies of many aquatic taxa (sea turtles, sharks, and marine mammals).
